# Anthraquinones from the Aerial Parts of *Rubia cordifolia* with Their NO Inhibitory and Antibacterial Activities

**DOI:** 10.3390/molecules27051730

**Published:** 2022-03-07

**Authors:** Han Luo, Wei Qin, Hong Zhang, Fu-Cai Ren, Wen-Tao Fang, Qing-Hua Kong, Liu Yang, Jian-Mei Zhang, Cheng-Wu Fang, Jiang-Miao Hu, Shou-Jin Liu

**Affiliations:** 1 College of Pharmacy, Anhui University of Chinese Medicine, Hefei 230011, China; luohan@stu.ahtcm.edu.cn (H.L.); weiqin@stu.ahtcm.edu.cn (W.Q.); zhanghong@ahtcm.edu.cn (H.Z.); fangwentao@ahtcm.edu.cn (W.-T.F.); cwfang1961@sina.com (C.-W.F.); 2 State Key Laboratory of Phytochemistry and Plant Resources in West China, Kunming Institute of Botany, Chinese Academy of Sciences, Kunming 650201, China; renfucai@ahmu.edu.cn (F.-C.R.); kongqinghua@mail.kib.ac.cn (Q.-H.K.); yangliu.8355@163.com (L.Y.); zhangjianmei7788@sina.com (J.-M.Z.); 3 College of Pharmacy, Anhui Medical University, Hefei 230011, China

**Keywords:** *Rubia cordifolia*, anthraquinones, NO inhibitory activity, antibacterial

## Abstract

The present study aimed to identify the composition of the aerial parts of *Rubia cordifolia* L. A chemical investigation on the EtOAc extracts from the aerial parts of *Rubia cordifolia* resulted in the isolation of four new anthraquinones, namely Cordifoquinone A–D (**1**–**4**), along with 16 known anthraquinones. Their structures were elucidated on the basis of NMR and HR-ESIMS data. All isolates were assessed for their inhibitory effects on NO production in LPS-stimulated RAW 264.7 macrophage cells. Compounds **1**, **3** and **10** exhibited significant inhibitory activities with IC_50_ values of 14.05, 23.48 and 29.23 μmol·L^−1^, respectively. Their antibacterial activities of four bacteria, *Escherichia coli* (ATCC 25922), *Staphylococcus aureus* subsp. *aureus* (ATCC 29213), *Salmonella enterica* subsp. *enterica* (ATCC 14028) and *Pseudomonas aeruginosa* (ATCC 27853), were also evaluated. Our results indicated that the antibacterial activity of these compounds is inactive.

## 1. Introduction

Infectious diarrhea (ID) is a kind of diarrhea caused by multiple pathogens and factors [1]. In 2012, a systematic analysis published in The Lancet showed that infectious diarrhea was the second leading cause of death in children under five years of age worldwide [2]. According to the pathogenesis, it can be divided into inflammatory diarrhea and secretory diarrhea, the former mainly caused by bacterial infection [3]. When the bacteria infect the human body, the pathogens further invade the intestinal mucosa and cause an inflammatory response that can lead to diarrhea. Nitric oxide (NO) is a bioactive molecule with extensive and important biological regulatory functions, which plays an important role in inflammation, tumors, and the cardiovascular system [4,5]. NO has been shown to be synthesized through the L-arginine/iNOS/NO pathway. In this pathway, NO and the amino acid L-citrulline are synthesized from the amino acid L-arginine by inducible nitric oxide synthase (iNOS) [4]. The excessive production of NO plays an important role in inflammatory response. Wink et al. found that when inflammation occurs, immune cells produce large amounts of inducible iNOS, which further produces NO for the immune response [6,7]. Therefore, inhibition of NO production is a direct indicator to verify the anti-inflammatory activity of compounds.

Worldwide, it is common for people to use medicinal plants to treat diarrhea [8,9,10]. Researchers have been working to find natural products with antidiarrheal properties from medicinal plants [11,12,13]. *Rubia cordifolia* Linn. is a grassy climbing vine herb that belongs to the genus Rubia and is found in China, India, Korea, Japan and the Far East of Russia, growing on sparse forests, forest margins and grasslands [14]. It is a well-known medicinal plant in Traditional Chinese Medicine (TCM). Rubiae Radix et Rhizoma, the dried root and rhizome of *R. cordifolia*, was first recorded in “Shennong Bencaojing” and used for hemorrhage syndrome and arthralgia. The aerial parts of *R. cordifolia* used for hemorrhage syndrome and diarrhea were recorded in “Lüchanyan Materia Medica”, another classic book on medicinal plants. In modern studies, *R. cordifolia* has been found to have a wide range of pharmacological activities. In 1983, Itokawa et al. found that a methanol extract of *R. cordifolia* had obvious anticancer activity [15]. In 2017, Gong et al. found that the water extract of *R. cordifolia* could treat senna leaf-induced diarrhea in mice [16]. In 2020, Wang et al. found that the ethanol extract of *R. cordifolia* could improve arachidonic acid metabolism by inhibiting COX-2 and cPLA2 [17]. Because of its excellent efficacy, *R. cordifolia* has been listed in the “Chinese Pharmacopeia” and is to this day [18]. Previous chemical studies revealed that the plant contains anthraquinones [19,20], naphthoquinones [20], terpenoids [21], cyclic hexapeptides [22] and lignans [23], some of which have antibacterial [24], anti-inflammatory [25] and antitumor activities [26].

At present, the research on *R. cordifolia* is mainly focused on the roots and rhizomes, and less on the aerial parts. Therefore, in order to find natural products with antibacterial or anti-inflammatory activities from this plant, we systematically studied the aerial parts of *R. cordifolia*. Our previous investigation identified eleven lignans from the aerial parts of *R. cordifolia* for the first time [23]. As a continuing investigation to find more undescribed and bioactive constituents from *R. cordifolia*, four new anthraquinones (**1**–**4**) along with 16 known anthraquinones (**5**–**20**) were isolated from the aerial parts of *R. cordifolia*. Compounds **1**–**5**, **9**, **10**, **13**, **15**–**17** and **20** were isolated from this plant for the first time. This study describes the isolation, structure elucidation, NO inhibitory and antibacterial assay of these anthraquinones.

## 2. Results and Discussion

**Figure 1:**
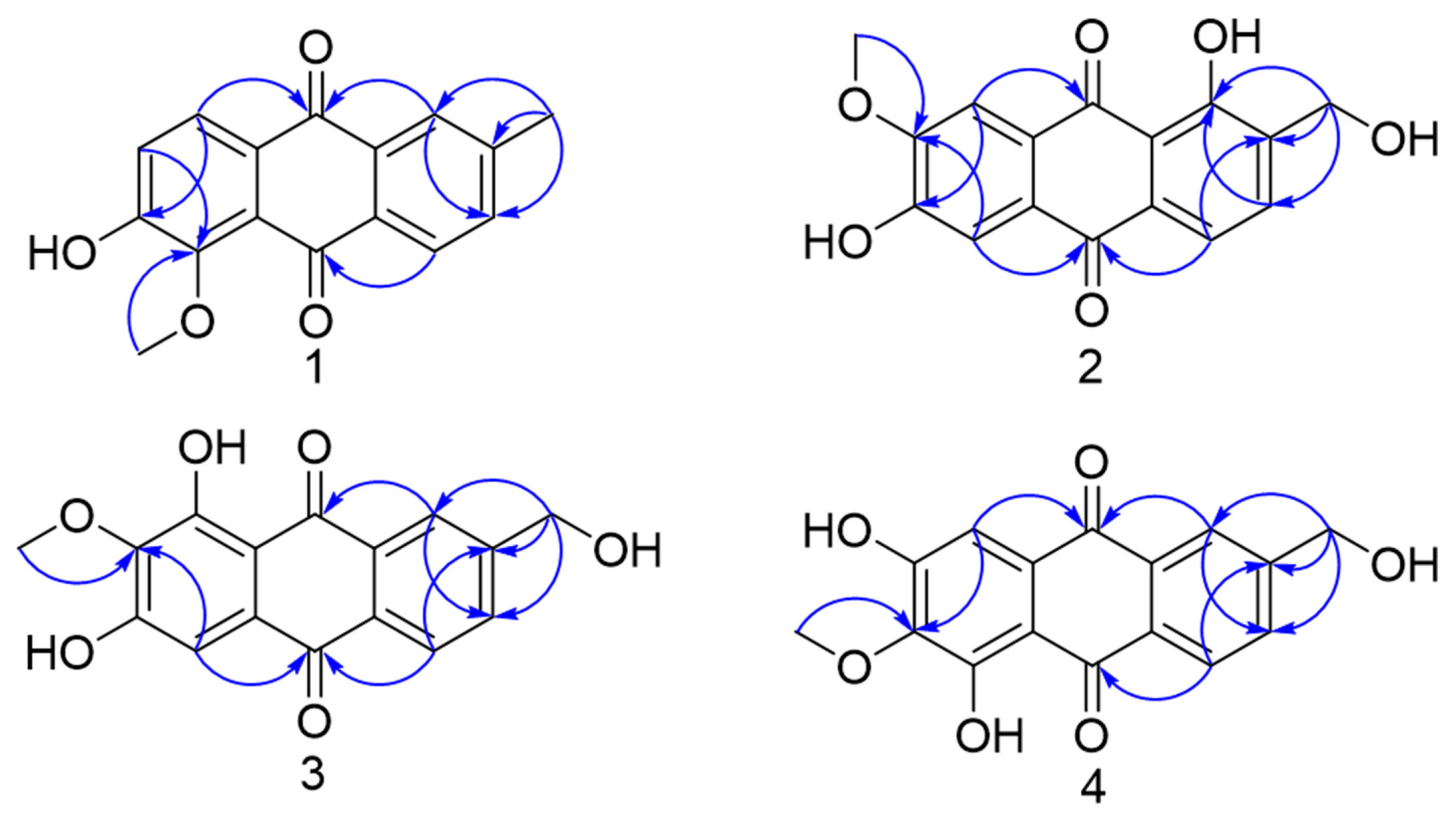
Key HMBC of compounds **1**–**4**.

Compound **1** was obtained as a yellow powder, and the molecular formula was determined as C_16_H_12_O_4,_ on the basis of HR-ESIMS (*m*/*z* 267.0669 [M − H]^−^, calcd. for C_16_H_11_O_4_, 267.0663). The UV spectrum showed maximum absorptions at 288 nm, suggesting a quinone structure. The ^1^H NMR data (Table 1) exhibited resonances which attributed to a 1, 3, 4-substituted benzene ring (δ_H_ 8.13 (1H, d, J = 7.9 Hz), 8.01 (1H, d, J = 1.8 Hz) and 7.71 (1H, dd, J = 7.9, 1.8 Hz)). The other benzene ring showed two orthodox-coupled aromatic proton resonances at (δ_H_ 8.04 (1H, d, J = 8.5 Hz) and 7.38 (1H, d, J = 8.5 Hz)). Moreover, aromatic methyl group δH 2.55 (3H, s), aromatic methoxy group δ_H_ 3.95 (3H, s) and phenolic hydroxyl group δ_H_ 9.40 (1H, s) protons were also observed. The ^13^C NMR exhibited 16 carbon signals, which consisted of 14 carbons of anthraquinone nucleus, a methoxy and a methyl. The HMBC (Figure 1) correlations of H-7 with C-5, 5-OMe with C-5 and H-8 with C-6/C-9 revealed that the methoxy group and hydroxyl group were connected at C-5 and C-6, respectively. In addition, the methyl group was deduced to be located at C-2 based on the HMBC correlations of 2-Me with C-1/C-2/C-3 and H-1 with C-3/C-9. Thus, the structure of compound **1** was established as 2-methyl-5-methoxy-6-hydroxyanthraquinone and named Cordifoquinone A, as shown in Figure 1.

Compound **2** was obtained as a yellow powder, and the molecular formula was determined to be C_16_H_12_O_6,_ on the basis of HR-ESIMS (*m*/*z* 299.0561 [M − H]^−^, calcd. for C_16_H_11_O_6_, 299.0560). The ^1^H NMR data (Table 1) exhibited two meta-coupled aromatic proton resonances at δ_H_ 7.79 (1H, d, J = 7.8 Hz) and 7.63 (1H, d, J = 7.8 Hz), two aromatic proton singlets at δ_H_ 7.54 and 7.44 (each 1H, s), an aromatic methine proton signals at δ_H_ 4.62 (2H, s), an aromatic methoxy group δ_H_ 3.95 (3H, s) and a phenolic hydroxyl group δ_H_ 13.03 (1H, s). The HMBC (Figure 1) correlations from the aromatic methine protons to C-1/C-2/C-3 and from H-4 to C-2/C-10, together with the ^1^H NMR data, implied the presence of a phenolic hydroxyl group at the C-1 and a hydroxymethyl group at the C-2 position. Moreover, the methoxy group was deduced to be located at C-7, and a phenolic hydroxyl group was deduced to be located at C-6, based on the HMBC correlations of the 7-OMe with C-7, H-5 with C-7/C-10 and H-8 with C-6/C-9. Therefore, the structure of compound **2** was confirmed as 1,6-dihydroxy-2-(hydroxymethyl)-7-methoxyanthraquinone and named Cordifoquinone B.

Compound **3** was obtained as a yellow powder and had the same molecular formula of C_16_H_12_O_6_ as compound **2,** based on HR-ESIMS (*m*/*z* 299.0561 [M − H]^−^, calcd. for C_16_H_11_O_6_, 299.0560). According to the NMR data (Table 1), **3** and **2** were found to have similar structures, except that the phenolic hydroxyl group on the hydroxymethyl side was moved to the methoxy side. The ^1^H NMR data exhibited resonances attributed to a 1, 3, 4-substituted benzene ring (δ_H_ 8.12 (1H, d, J = 1.7 Hz), 8.06 (1H, d, J = 7.9 Hz) and 7.76 (1H, dd, J = 7.9, 1.7 Hz)), an aromatic proton singlet at δ_H_ 7.19 (1H, s), an aromatic methine proton signals at δ_H_ 4.67 (2H, s), an aromatic methoxy group δ_H_ 3.83 (3H, s) and an aromatic hydroxyl group δ_H_ 13.03 (1H, s). The methoxy group was deduced to be located at C-7 based on the HMBC (Figure 1) correlations of 7-OMe with C-7 and H-5 with C-7/C-10. Moreover, the HMBC correlations from the aromatic methine protons to C-1/C-2/C-3, from H-4 to C-2/C-10 and from H-1 to C-9 suggested that the hydroxymethyl group was directly connected to C-2. Based on the data mentioned above, the structure of compound **3** was defined to be 6,8-dihydroxy-2-(hydroxymethyl)-7-methoxyanthraquinone and named Cordifoquinone C.

Compound **4** was obtained as a yellow powder and had the same molecular formula of C_16_H_12_O_6_ as compound **3,** based on HR-ESIMS (*m*/*z* 299.0561 [M − H]^−^, calcd for C_16_H_11_O_6_, 299.0560). Its ^1^H and ^13^C NMR data (Table 1) were very similar to those of compound **3**. The difference between them was that two phenolic hydroxyl groups and the methoxy group position of compound **4** were connected to C-5/C-7 and C-6, respectively, which were supported by the HMBC (Figure 1) correlations from the aromatic methine protons to C-1/C-2/C-3, H-1 to C-9/C-3, 6-OMe to C-6 and H-8 to C-6/C-9. Accordingly, the whole structure of compound **4** was finally determined as 5,7-dihydroxy-2-(hydroxymethyl)-6-methoxyanthraquinone and named Cordifoquinone D.

**Scheme 1:**
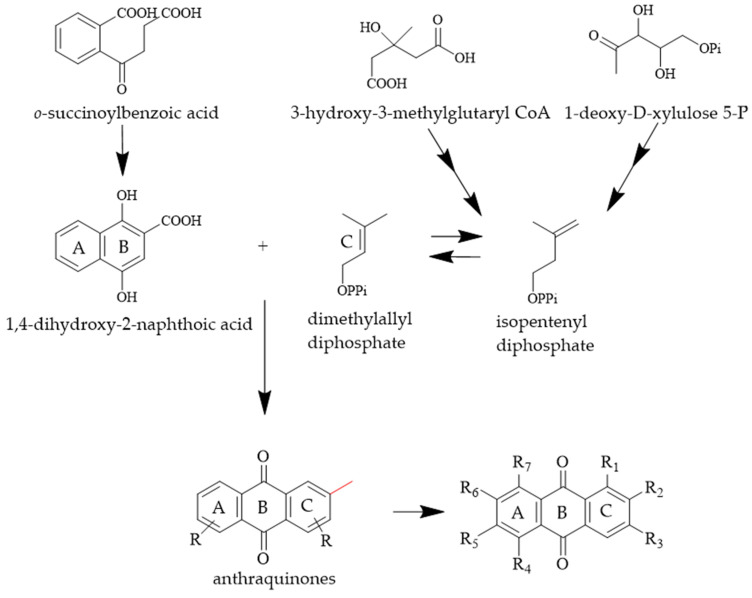
Plausible biosynthetic pathway for compounds **1**–**20**.

Additionally, 16 known anthraquinones, pustuline [27], 5,7-dihydroxy-6-methoxy-2-methylanthraquinone [28], soranjidiol [29], 1,6-dihydroxy-5-methoxy-2-methylanthraquinone [29], 1,5-dihydroxy-6-methoxy-2-methylanthraquinone [29], copareolatin 6-methylether [30], rubiadin [29], 2-methyl-1,3,6-trihydroxyanthraquinone [19], 7-hydroxy-2-(hydroxymethyl)-6-methoxyanthraquinone [31], 1-hydroxy-2-(hydroxymethyl)anthraquinone [32], 1,6-dihydroxy-2-(hydroxymethyl)-5-methoxyanthraquinone [33], 1,6-dihydroxy-2-(hydroxymethyl)-5,7-dimethoxyanthraquinone [34], 1,3-dihydroxy-2-hydroxymethyl-6-methoxyanthraquinone [35], 1,3-dihydroxy-2-(hydroxymethyl)-5,6-dimethoxyanthraquinone [36], 3,6-dihydroxy-2-(hydroxymethyl)-1-methoxyanthraquinone [37] and alizarin 1-methyl ether [38] were isolated from the aerial parts of *R. cordifolia*, as shown in Table 2. Their structures were elucidated by comparison of the NMR and ESI data with those reported in the literature. It is worth mentioning that anthraquinones in *R. cordifolia* are considered to be of the Rubia type; rings A and B are derived from horismite and α-ketoglutarate [39,40], whereas ring C is formed from isopentenyl diphosphate (IPP) [41]. Anthraquinone biosynthesized by this pathway has a methylation site in the C ring [42]. The aryl methyl group at site 2 can be further oxidized to hydroxymethyl. So, we hypothesized that compounds **1**–**20** might be synthesized by this pathway, as shown in Scheme 1.

All isolated compounds **1**–**20** were evaluated for their inhibitory effects on NO production in LPS-activated RAW264.7 macrophage cells. Cell viability was first examined by MTT assay to exclude false positive results caused by the potential cytotoxicity of the tested compounds. NG-Methyl-L-arginine acetate salt, an inhibitor of NO synthase, was used as a positive control (IC_50_ 42.36 ± 2.47 μmol·L^−1^). As a result, compounds **1**, **3** and **10** showed significant inhibitory effects against NO production, with IC_50_ values of 14.05 ± 0.48, 23.48 ± 1.05 and 29.23 ± 0.34 μmol·L^−1^ (Table 3), respectively, which were more potent than the positive control. By comparing the structure and activity of compounds **1**, **3**, **10**, we found that the orthoposition of methoxyl and phenolic hydroxyl groups in the A ring of anthraquinone was essential for the NO inhibitory activity, and the hydroxyl group at position C-6 or C-7 provided a greater contribution. It is known that the synthesis of NO requires the addition of superoxide anions [43]. So, we hypothesized that the hydroxyl group in anthraquinone may capture superoxide anions, which are necessary for the synthesis of NO. Compound **1** showed stronger NO inhibitory activity than compounds **3** and **10**, indicating that the activity was not necessarily related to the number of hydroxyl groups but may be related to the position of hydroxyl groups. We hypothesized that it may be due to the formation of intramolecular hydrogen bonds between hydroxyl group at position C-5 or C-8 and ketone carbonyl group at position C-9 or C-10, which makes the compound structure more stable and reduces its ability to capture superoxide anions. Through this speculation, it is reasonable to explain why compound **3** showed slightly higher NO inhibitory activity than compound **10**. In addition, all compounds were evaluated for their antibacterial activities against four bacteria: Escherichia coli (ATCC 25922), Staphylococcus aureus subsp. aureus (ATCC 29213), Salmonella enterica subsp. enterica (ATCC 14028), and Pseudomonas aeruginosa (ATCC27853). However, no antibacterial activity was observed for these compounds at concentrations below 128 μg·mL^−1^ in this bioassay.

## 3. Experimental

### 3.1. General Experimental Procedures

UV spectra were taken on a Shimadzu UV-2401 spectrophotometer (Shimadzu, Kyoto, Japan). IR (KBr) spectra were recorded on a Bruker Vertex 70 (Bruker, Bremerhaven, Germany). The 1D and 2D NMR experiments were carried out using Avance III-400 and III-600 spectrometers (Bruker, Bremerhaven, Germany) with TMS as an internal standard. HR-ESIMS data were recorded on an Agilent 6540 Q-TOF mass spectrometer (Agilent Technologies, Santa Clara, CA, USA). Silica gel (200–300 mesh, Linyi Haixiang Co. Ltd., Linyi, China) and Sephadex LH-20 gel (Amersham Biosciences, Uppsala, Sweden) were employed for column chromatography. Thin-layer chromatography (TLC) analyses were performed on silica gel GF254 plates (Haohong, Chemical Co. Ltd., Yantai, China). HPLC purification was achieved on an Agilent 1100 instrument (Agilent Technologies, Santa Clara, CA, USA) with an Agilent ZORBAX SB-C18 column (5 μm, 9.4 × 250 mm, Agilent Technologies, Santa Clara, CA, USA).

### 3.2. Plant Materials

The aerial parts of *Rubia cordifolia* Linn. Were collected in June 2020 from Huaiyuan County, Anhui Province, China, and authenticated by Prof. Shou-Jin Liu from Anhui University of Traditional Chinese Medicine (Anhui, China). A voucher specimen (No. 20200601QC) was deposited at the Chinese Medicine Resource Center, Anhui University of Traditional Chinese Medicine (ACM).

### 3.3. Extraction and Isolation

**Figure 2:**
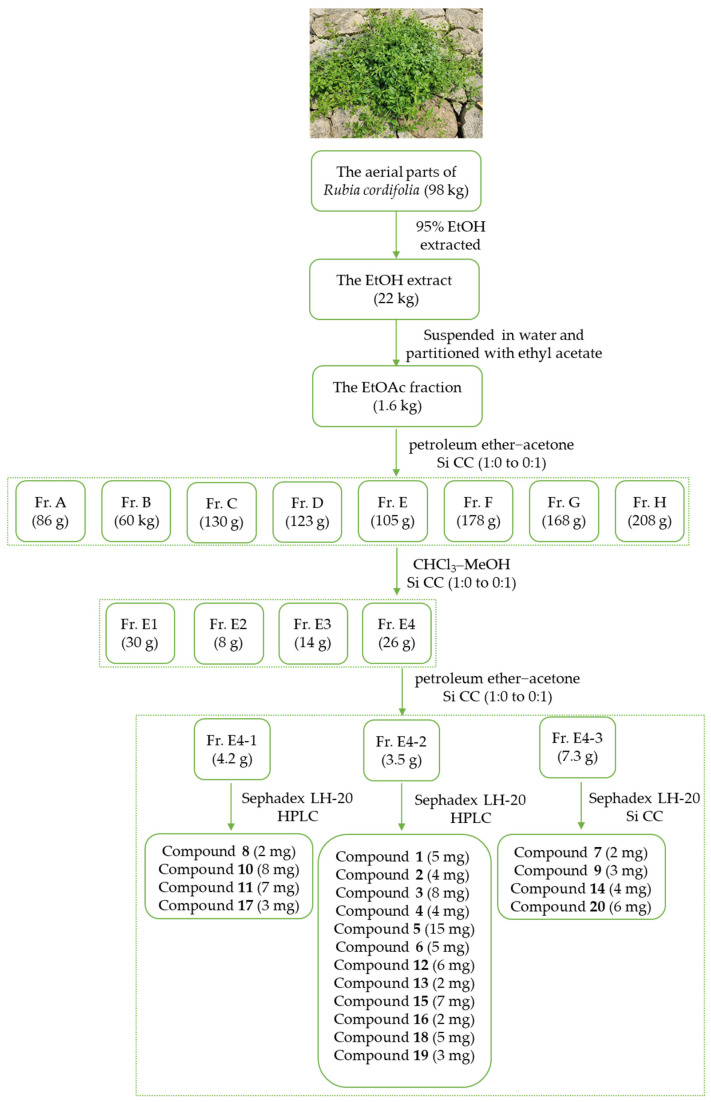
The extraction and separation process of the aerial parts of *Rubia cordifolia* L.

The air-dried and powdered aerial parts of *R. cordifolia* (22 kg) were extracted with 95% EtOH at room temperature. After removal of the solvent under reduced pressure, the EtOH extract (22 kg) was suspended in water and partitioned with EtOAc. The EtOAc fraction (1.6 kg) was dissolved in EtOAc and chromatographed on silica gel column chromatography (Si CC), eluted with petroleum ether−acetone (1:0 to 0:1) to yield eight fractions, Fr. A–H (86 g, 60 g, 130 g, 123 g, 105 g, 178 g, 168 g, and 208 g). Fr. E (105 g) was dissolved in acetone and subjected to Si CC eluted with a gradient of CHCl**_3_**–MeOH (1:0 to 0:1) to obtain four major fractions, Fr. E1 to Fr. E4 (30 g, 8 g, 14 g, and 26 g). The extraction and separation process is shown in Figure 2.

Fr. E4 (26 g) was dissolved in acetone and further chromatographed over Si CC using petroleum ether−acetone (1:0 to 0:1) to give three subfractions, Fr. E4-1 to Fr. E4-3 (4.2 g, 3.5 g, 7.3 g). Compounds **8** (2 mg), **10** (8 mg), **11** (7 mg) and **17** (3 mg) were purified from Fr. E 4-1 (4.2 g) by Sephadex LH-20 (MeOH-CHCl_3_, 1:1) and semipreparative HPLC (H_2_O-MeOH, 75:25, 2.0 mL/min). Fr. E 4-2 (3.5 g) was applied to Sephadex LH-20 (CHCl_3_-MeOH, 1:1) to yield four subfractions (Fr. E4-2-1 to Fr. E4-2-4). Fr. E4-2-2 (127 mg) was separated by semipreparative HPLC (H_2_O-MeOH, 70:30, 2.0 mL·min^−1^) to obtain 12 (6 mg) and 18 (5 mg). Fr. E4-2-3 (238 mg) was separated by semipreparative HPLC (H_2_O-MeOH, 60:40, 2.0 mL·min^−1^) to afford 1 (5 mg), 2 (4 mg), 3 (8 mg), 5 (15 mg) and 15 (7 mg). Fr. E4-2-4 (182 mg) was separated by semipreparative HPLC (H_2_O-MeOH, 60:40, 2.0 mL·min^−1^) to afford 4 (4 mg), 6 (5 mg), 13 (2 mg), 16 (2 mg) and 19 (3 mg). Fr. E4-3 (7.3 g) gave 7 (2 mg), 9 (3 mg), 14 (4 mg) and 20 (6 mg) after repeated chromatography over Sephadex LH-20 (CHCl_3_-MeOH, 1:1) and Si CC gradient of CHCl_3_-MeOH (1:0 to 0:1).

Cordifoquinone A (1): Yellow powder; UV (MeOH) λ_max_ (log *ε*) 251 (4.01), 264 (3.97) nm; IR (KBr) ν_max_ 3423, 2955, 1666, 1629, 1581, 1428, 1363, 1302, 1256, 1024, 799 cm^−1^; ^1^H and ^13^C NMR data, see Table 1 and Figure S1; HR-ESIMS (*m*/*z* 267.0669 [M − H]^−^, calcd. for C_16_H_11_O_4_, 267.0663).

Cordifoquinone B (2): Yellow powder; UV (MeOH) λ_max_ (log *ε*) 288 (3.89), 249 (3.53) nm; IR (KBr) ν_max_ 3433, 2936, 1632, 1591, 1518, 1424, 1384, 1308, 1221, 1116, 1064, 754 cm^−1^; ^1^H and ^13^C NMR data, see Table 1 and Figure S2; HR-ESIMS (*m*/*z* 299.0561 [M − H]^−^, calcd. for C_16_H_11_O_4_, 299.0560).

Cordifoquinone C (3): Yellow powder; UV (MeOH) λ_max_ (log *ε*) 248 (3.74), 285 (3.73) nm; IR (KBr) ν_max_ 3443, 2952, 1662, 1632, 1596, 1452, 1385, 1340, 1279, 1219, 1134, 988 cm^−1^; ^1^H and ^13^C NMR data, see Table 1 and Figure S3; HR-ESIMS (*m*/*z* 299.0561 [M − H]^−^, calcd. for C_16_H_11_O_6_, 299.0560).

Cordifoquinone D (4): Yellow powder; UV (MeOH) λmax (log *ε*) 248 (3.47), 285 (3.41) nm; IR (KBr) ν_max_ 3439, 2926, 1633, 1601, 1577, 1384, 1282, 1162, 975, 851 cm^−1^; ^1^H and ^13^C NMR data, see Table 1 and Figure S4; HR-ESIMS (*m*/*z* 299.0561 [M − H]^−^, calcd. for C_16_H_11_O_6_, 299.0560).

### 3.4. NO Inhibitory Assay

Murine macrophage cell line RAW264.7 was obtained from Shanghai Cell Bank of Chinese Academy of Sciences. RAW264.7 cells were seeded in 96-well cell culture plates (1.5 × 10^5^ cells/well), stimulated with 1 μg·mL^−1^ LPS (Sigma, St. Louis, MO, USA) for 18 h, and treated with serial dilutions of the compounds with a maximum concentration of 50 μM in triplicate during this period. NG-Methyl-L-arginine acetate salt (L-NMMA, Sigma), a well-known nitric oxide synthase (NOS) inhibitor, was used as a positive control [44]. NO production in the supernatant was assessed by Griess reagents (Reagent A and Reagent B, respectively, Sigma). The absorbance at 570 nm was measured with a microplate reader (Thermo, Waltham, MA, USA). The viability of RAW264.7 cells was evaluated by the MTS assay simultaneously to exclude the interference of the cytotoxicity of the test compounds. All tests were performed in triplicate, and the results were expressed as IC_50_ values.

### 3.5. Antibacterial Assay

*Escherichia coli* (ATCC 25922), *Staphylococcus aureus* subsp. *aureus* (ATCC 29213), *Sal-monella enterica* subsp. *enterica* (ATCC 14028), and *Pseudomonas aeruginosa* (ATCC 27853) were obtained from China General Microbiological Culture Collection Center. The inoculated strains and suspension were cultured for 24 h and adjusted to 0.5 McFarlane standard turbidity. A 96-well plate was prepared by injecting 100 µL of Muller Hinton broth/Luria-Bertani into each well. A volume of 100 µL of stock solution from the initial prepared samples was added to the first well. Then, 100 µL from their two-fold serial dilutions were transferred into consecutive wells, and 100 µL of the inoculums was added to achieve a final inoculum concentration of 5 × 10^5^ CFU/mL. The final volume in each well was 200 µL. Ceftazidime (Yuanye Co. Ltd., Shanghai, China) and sodium penicillin G (Labgic Co. Ltd., Beijing, China) were used as positive controls. After incubation at 37 °C for 24 h, growth was monitored by a Microplate Reader at 625 nm (PowerWave XS, BioTek, Winooski, VT, USA) [45].

## 4. Conclusions

Four new anthraquinones (**1**–**4**) and 16 known anthraquinones (**5**–**20**) were isolated from a 95% EtOH extract of aerial prat from *R. cordifolia* and elucidated by UV, IR, HR-ESIMS and NMR spectroscopic data. Among the isolated compounds, compounds **1**, **3** and **10** displayed NO inhibitory activity with IC_50_ values of 14.05, 23.48 and 29.23 μmol·L^−1^. The structure and activity relationship analysis revealed that the orthoposition of methoxyl and phenolic hydroxyl groups in the A ring of anthra-quinone was essential for the NO inhibitory activity. The hydroxyl group at position C-6 or C-7 may have promoted the NO inhibitory activity, whereas the hydroxyl group at position C-5 or C-8 had the opposite effect. In addition, this series of anthraquinones showed no activity in tests of antibacterial activity. This study proved the reasonableness of using the aerial part of *R. cordifolia* as a medicine to treat diarrhea in Traditional Chinese medicine. It also provides scientific evidence and a foundation for the understanding of the anti-inflammatory effects and further utilization of *R. cordifolia*. In future research, both the extract and the natural products of *R. cordifolia* can be considered a drug or a health supplement to treat diarrhea.

## Figures and Tables

**Table 1:** ^1^H and ^13^C NMR spectroscopic data (*δ* in ppm, *J* in Hz) for compounds **1**–**4**.

No	1 ^a^		2 ^a^		3 ^b^		4 ^a^	
	*δ*_H_	*δ*_C_	*δ*_H_	*δ*_C_	*δ*_H_	*δ*_C_	*δ*_H_	*δ*_C_
1	8.01, d (1.8)	127.2		158.6	8.12, d (1.7)	123.5	8.06, d (1.7)	124.0
2		145.5		138.3		149.8		149.5
3	7.71, dd (7.9, 1.8)	135.4	7.63, d (7.8)	133.1	7.76, dd (7.9, 1.7)	131.6	7.78, dd (7.9, 1.7)	131.7
4	8.13, d (7.9)	127.8	7.79, d (7.8)	118.8	8.06, d (7.9)	126.8	8.13, d (7.9)	126.3
4 ^a^		133.5		131.8		131.7		132.1
5		148.4	7.54, s	113.4	7.19, s	109.9		157.0
6		158.1		155.1		157.0		139.8
7	7.38, d (8.5)	121.6		153.5		139.6		160.3
8	8.04, d (8.5)	125.8	7.44, s	108.8		159.2	7.16, s	110.5
8 ^a^		127.9		125.2		109.6		129.3
9		182.6		187.8		186.1		181.9
9 ^a^		133.7		115.2		133.2		132.9
10		182.7		181.8		181.4		185.5
10 ^a^		127.5		129.2		129.1		109.0
2-Me	2.53, s	21.6						
2-CH_2_OH			4.62, s	57.9	4.67, s	62.2	4.66, s	62.2
5-OMe	3.95, s	61.7						
6-OMe							3.82, s	59.8
7-OMe			3.95, s	56.3	3.83, s	59.9		

^a 1^H at 400 MHz and ^13^C at 150 MHz in Acetone-*d_6_*. ^b 1^H at 400 MHz and ^13^C at 150 MHz in DMSO-*d_6_*.

**Table 2:** Chemical structures of isolated compounds **1**–**20** from *Rubia cordifolia*.

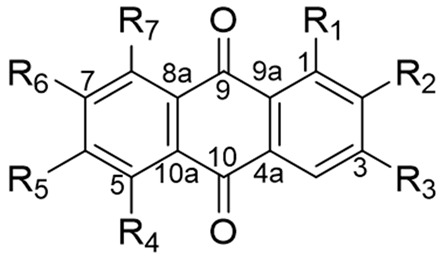							
	R_1_	R_2_	R_3_	R_4_	R_5_	R_6_	R_7_
**1**	H	Me	H	OMe	OH	H	H
**2**	OH	CH_2_OH	H	H	OH	OMe	H
**3**	H	CH_2_OH	H	H	OH	OMe	OH
**4**	H	CH_2_OH	H	OH	OMe	OH	H
**5**	H	Me	H	H	OMe	OH	H
**6**	H	Me	H	OH	OMe	OH	H
**7**	OH	Me	H	H	OH	H	H
**8**	OH	Me	H	OMe	OH	H	H
**9**	OH	Me	H	OH	OMe	H	H
**10**	OH	Me	H	OH	OMe	OH	H
**11**	OH	OMe	OH	H	H	H	H
**12**	OH	OMe	OH	H	OH	H	H
**13**	H	CH_2_OH	H	H	OMe	OH	H
**14**	OH	CH_2_OH	H	H	H	H	H
**15**	OH	CH_2_OH	H	OMe	OH	H	H
**16**	OH	CH_2_OH	H	OMe	OH	OMe	H
**17**	OH	CH_2_OH	OH	H	OMe	H	H
**18**	OH	CH_2_OH	OH	OMe	OMe	H	H
**19**	OMe	CH_2_OH	OH	H	OH	H	H
**20**	OMe	OH	H	H	H	H	H

**Table 3:** NO inhibitory activities of compounds **1**, **3** and **10**.

Compounds	NO Inhibitory Effects (IC_50_/μmol·L^−1^)	RAW 264.7 Cell Viability ^a^ (%)
**1**	14.05 ± 0.48	105.69 ± 0.25
**3**	23.48 ± 1.05	97.67 ± 1.21
**10**	29.23 ±0.34	101.80 ± 1.10
L-NMMA ^b^	42.36 ± 2.47	98.72 ± 0.94

^a^ RAW 264.7 cells treated with samples at 50 μmol·L^−1^. ^b^ Positive control.

## Data Availability

The data presented in this study are available in the Supplementary Materials.

## Supplementary Materials

The following supporting information can be downloaded online, Figure S1: ^1^H, ^13^C and HMBC NMR spectra of compound **1**; Figure S2: ^1^H, ^13^C and HMBC NMR spectra of compound **2**; Figure S3: ^1^H, ^13^C and HMBC NMR spectra of compound **3**; Figure S4: ^1^H, ^13^C and HMBC NMR spectra of compound **4**.

## Sample Availability

Samples of the compounds compound **1** to **20** are available from the authors.

## Abbreviations

ID	infectious diarrhea
NO	nitric oxide
iNOS	inducible nitric oxide synthase
TCM	Traditional Chinese Medicine
HR-ESIMS	high resolution electrospray ionization mass spectroscopy
UV	ultraviolet-visible
NMR	nuclear magnetic resonance
HMBC	heteronuclear multiple bond correlation
MTT	3-(4,5-dimethylthiazol-2-yl)-2,5-diphenyltetrazolium bromid
IR	infrared ra
Q-TOF	quadrupole-time of flight
HPLC	high performance liquid chromatography
TLC	thin-layer chromatography
Si CC	silica gel column chromatography
EtOH	ethyl alcohol
EtOAc	ethyl acetate
MeOH	Methanol
USA	The United States of America
