# Supplementary material for: Anthraquinones from the Aerial Parts of Rubia cordifolia with Their NO Inhibitory and Antibacterial Activities
Source: Molecules. 2022 Mar 7;27(5):1730. doi: 10.3390/molecules27051730 (PMC8911942; doi:10.3390/molecules27051730)
Supplement: Supplementary file 1 [file molecules-27-01730-s001.zip › molecules-1588149-supplementary.pdf]

Anthraquinones from the aerial parts of Rubia cordifolia with their NO inhibitory and antibacterial activities

Han Luo[1], Wei Qin[1], Hong Zhang[1], Fu-Cai Ren[3], Wen-Tao Fang[1], Qing-Hua Kong[2], Jian-Mei Zhang[2], Cheng-Wu Fang[1], Jiang-Miao Hu[2*], Shou-Jin Liu[1*]

[1] *College of Pharmacy, Anhui University of Chinese Medicine, Hefei 230011, China;*

[2] *State Key Laboratory of Phytochemistry and Plant Resources in West China, Kunming Institute of Botany, Chinese Academy of Sciences, Kunming 650201, China.*

[3] *College of Pharmacy, Anhui Medical University, Hefei 230011, China.*

[*Corresponding author] E-mails: hujiangmiao@mail.kib.ac.cn (Hu Jiang-Miao); shjinliu@sina.com (Liu Shou-Jin)

List of supplementary material Pages

**Figure S1**	^{1}H, ^{13}C and HMBC NMR spectra of compound **1**	1
**Figure S2**	^{1}H, ^{13}C and HMBC NMR spectra of compound **2**	2
**Figure S3**	^{1}H, ^{13}C and HMBC NMR spectra of compound **3**	4
**Figure S4**	^{1}H, ^{13}C and HMBC NMR spectra of compound **4**	5

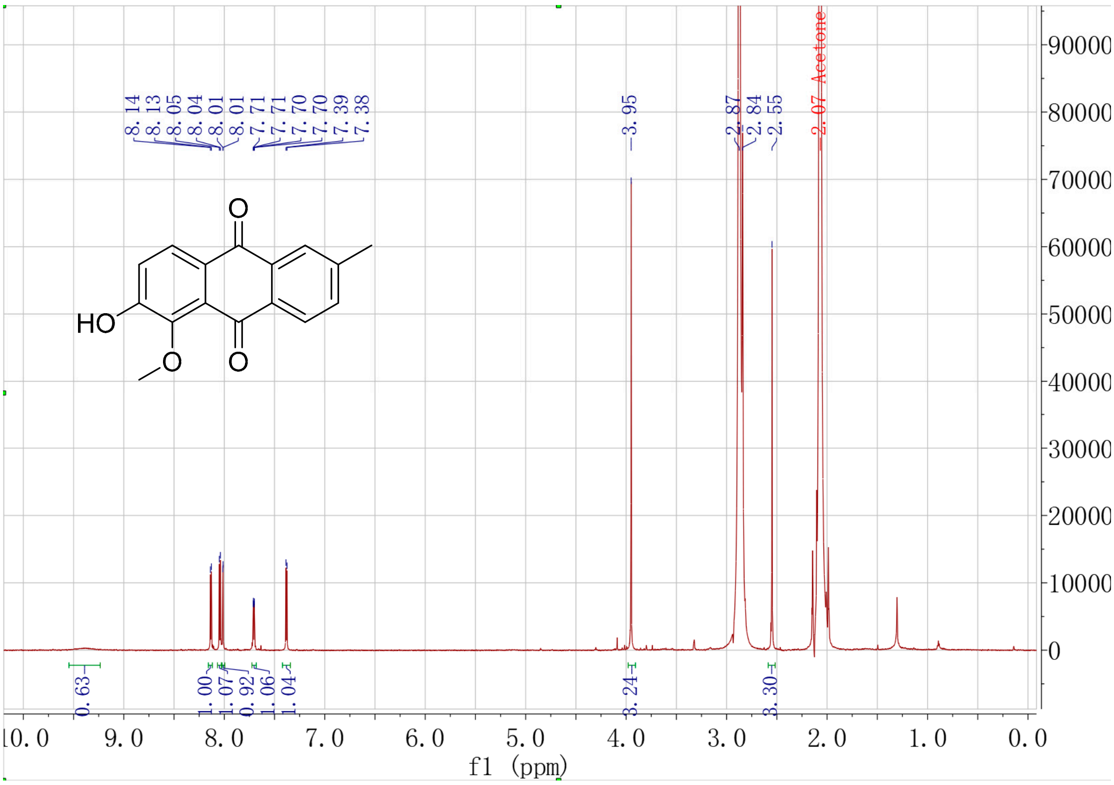

Figure S1-1. ^{1}H NMR spectrum of compound **1**

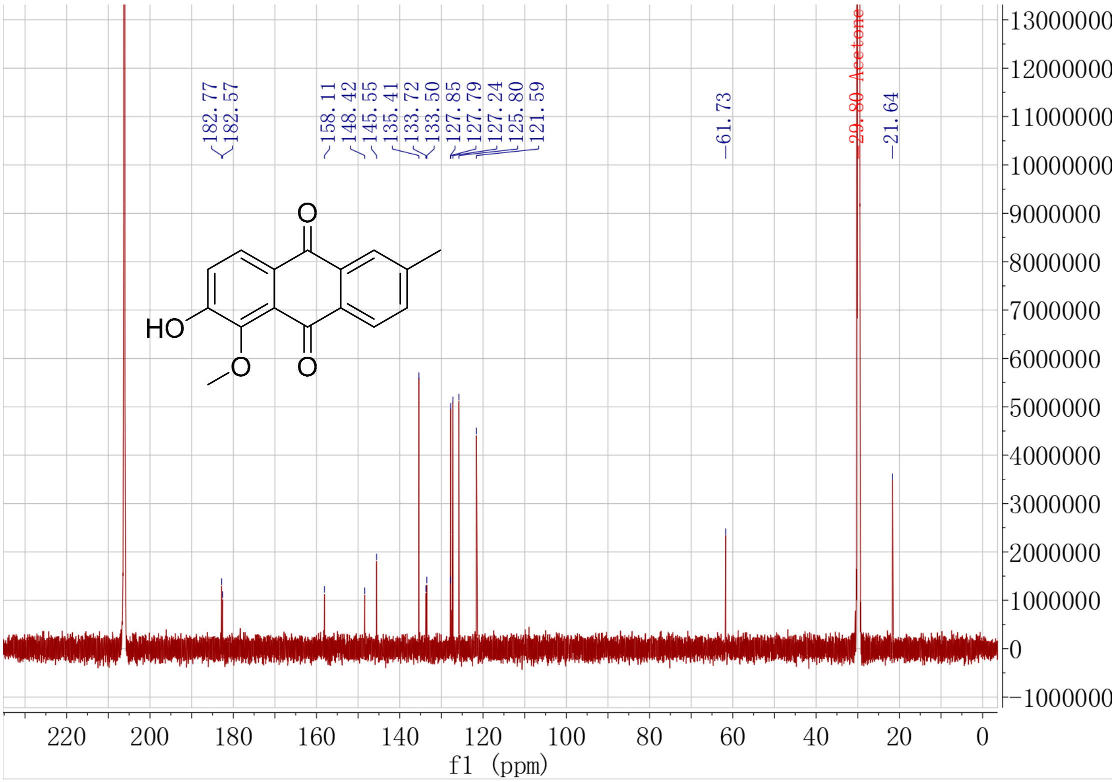

Figure S1-2. ^{13}C NMR spectrum of compound **1**

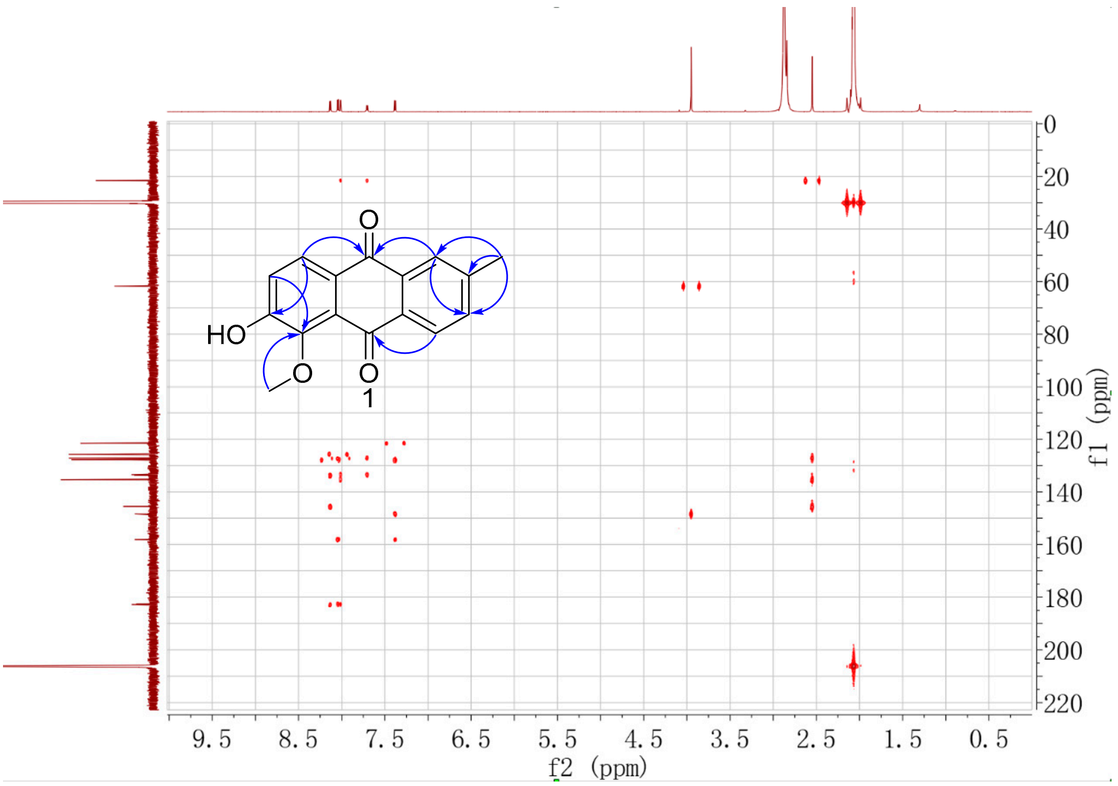

Figure S1-3. HMBC NMR spectrum of compound **1**

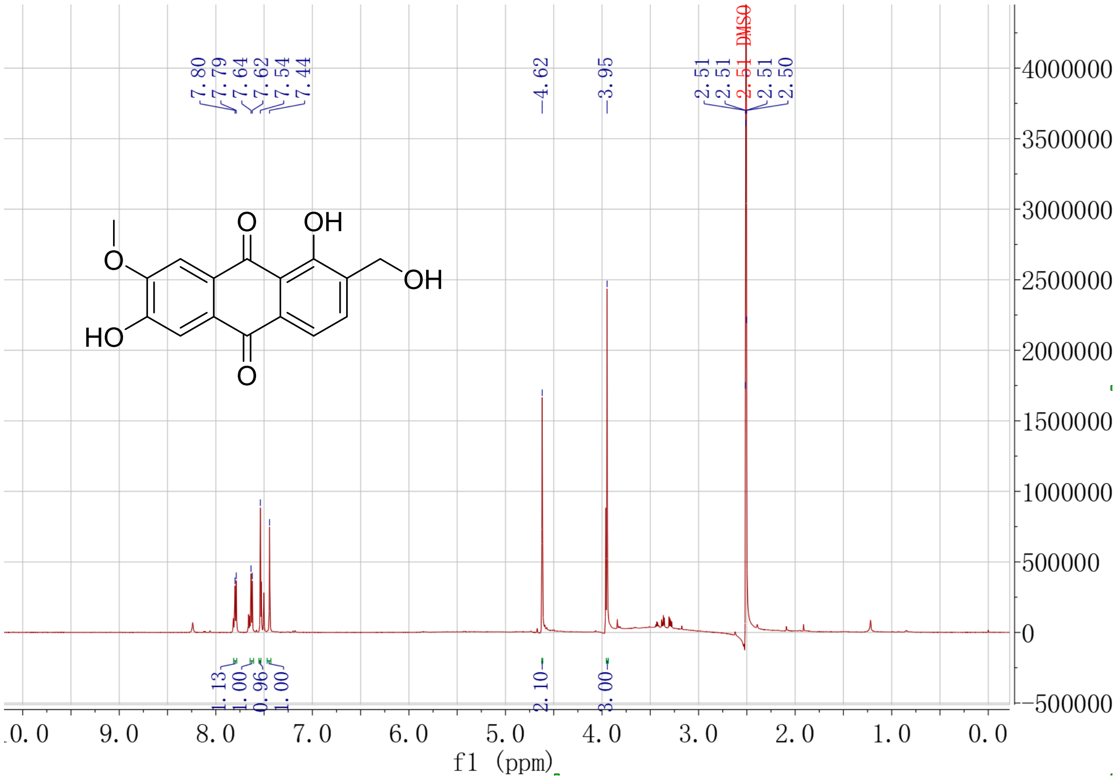

Figure S2-1. ^{1}H NMR spectrum of compound **2**

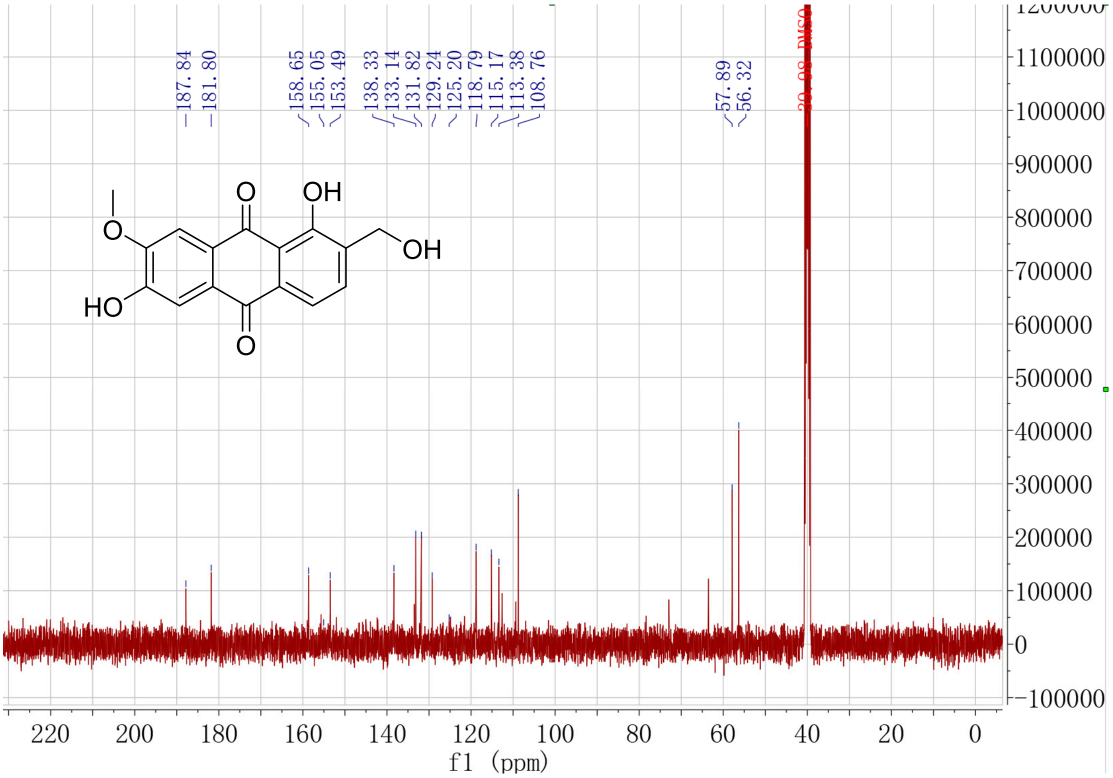

Figure S2-2. ^{13}C NMR spectrum of compound **2**

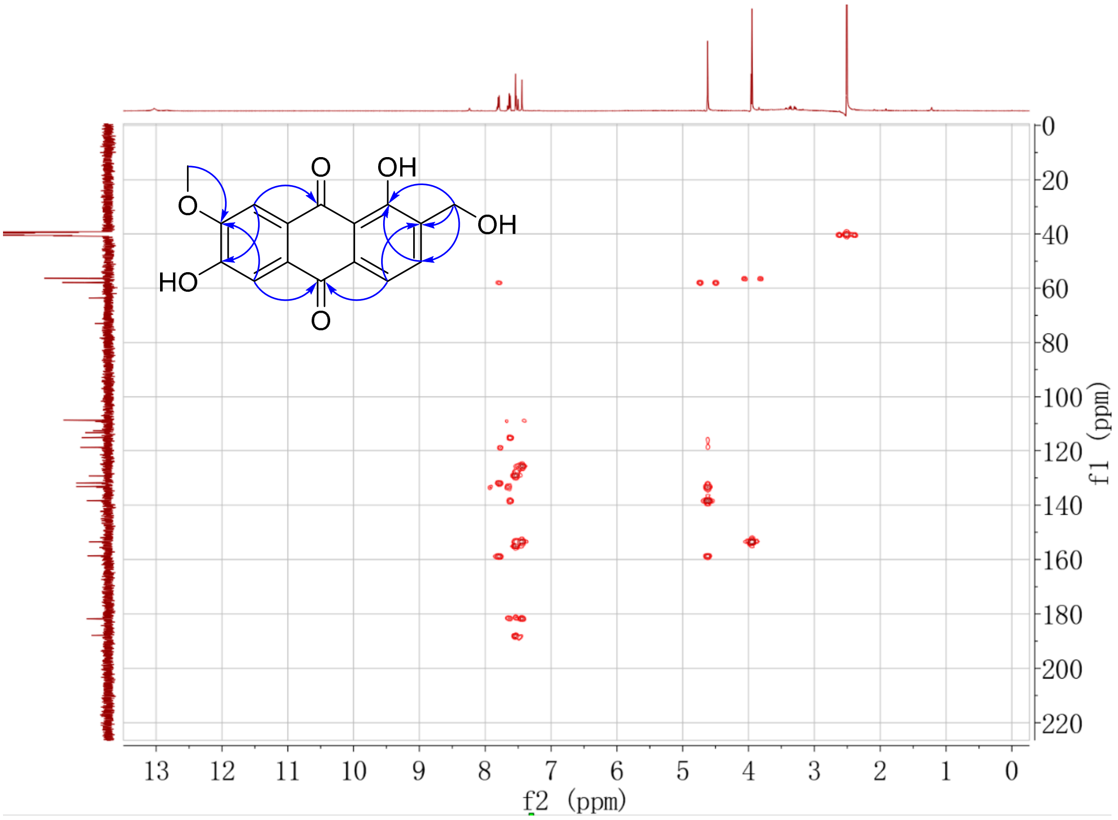

Figure S2-3. HMBC NMR spectrum of compound **2**

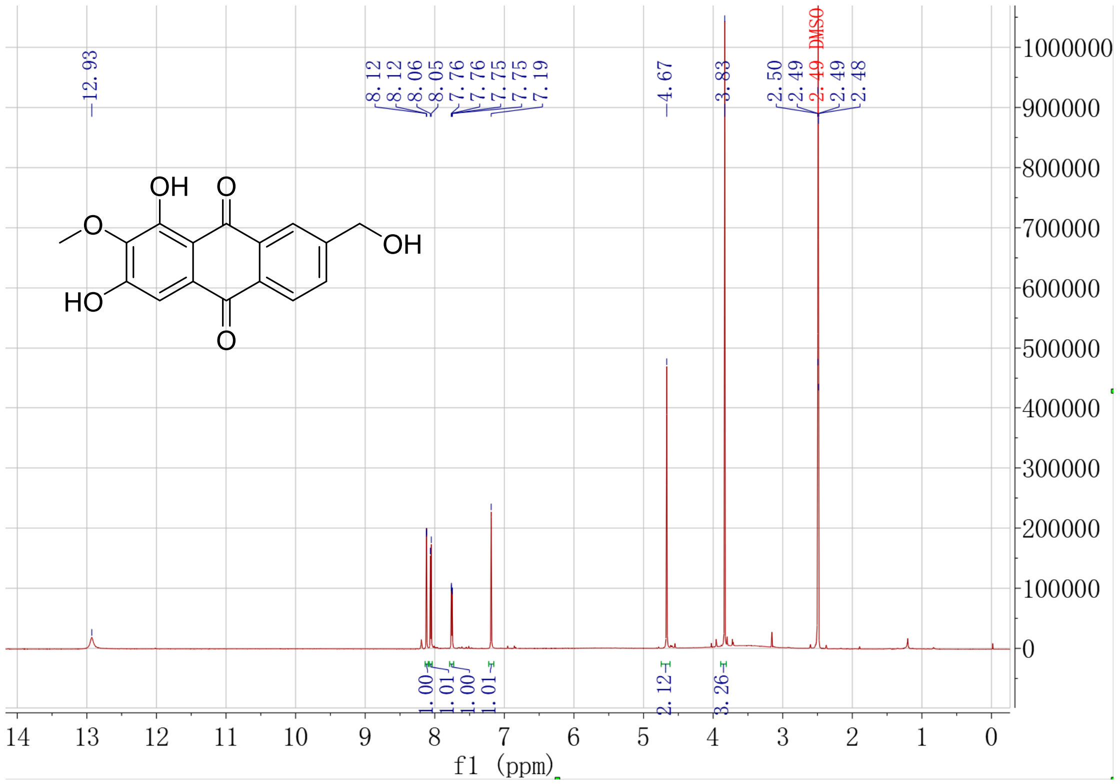

Figure S3-1. ^{1}H NMR spectrum of compound **3**

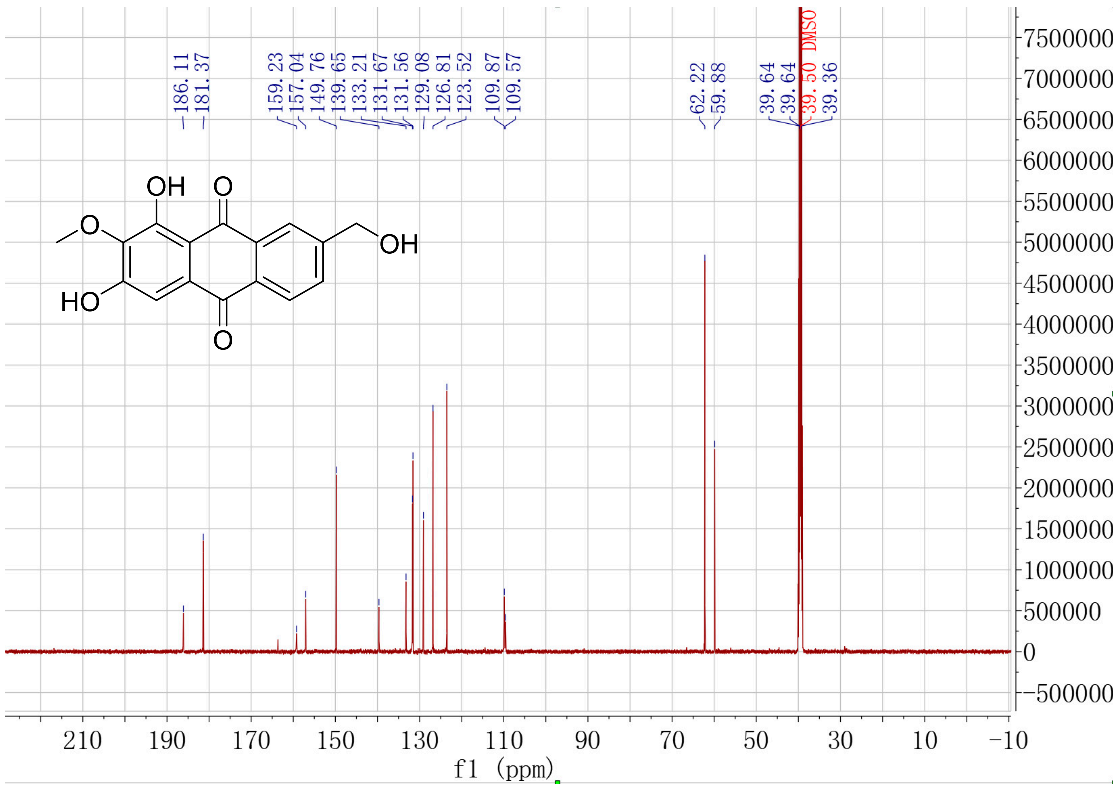

Figure S3-2. ^{13}C NMR spectrum of compound **3**

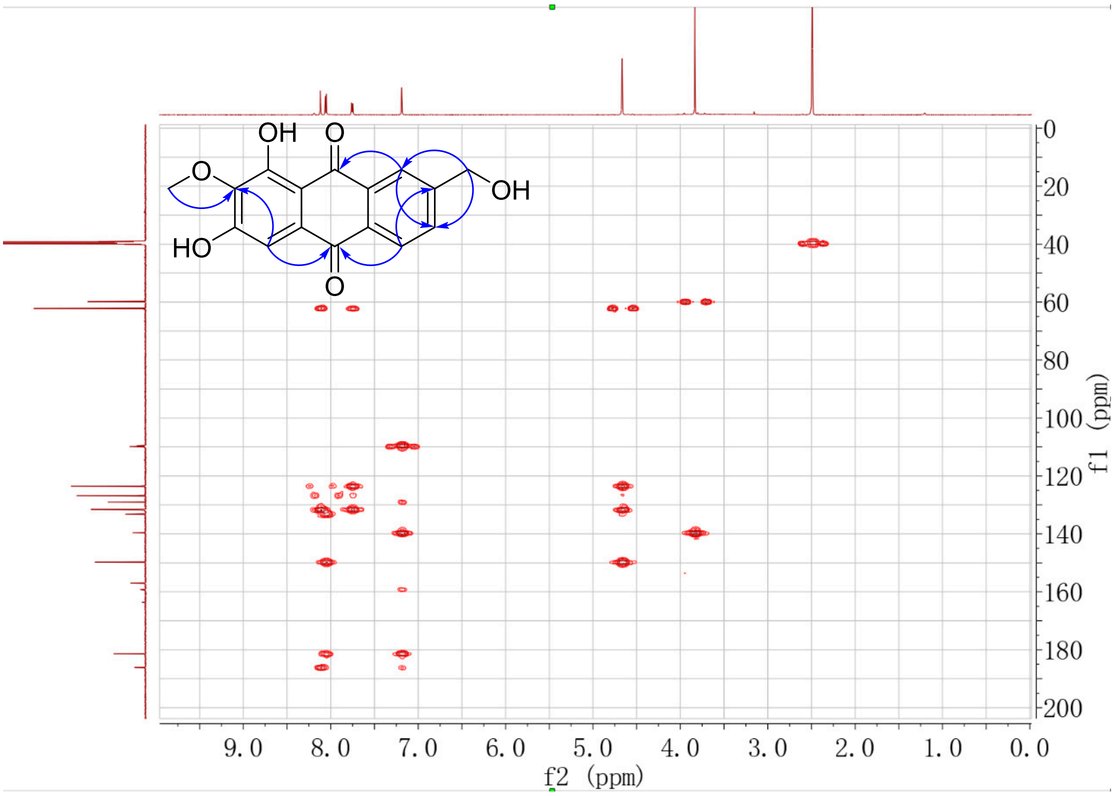

Figure S3-3. HMBC NMR spectrum of compound **3**

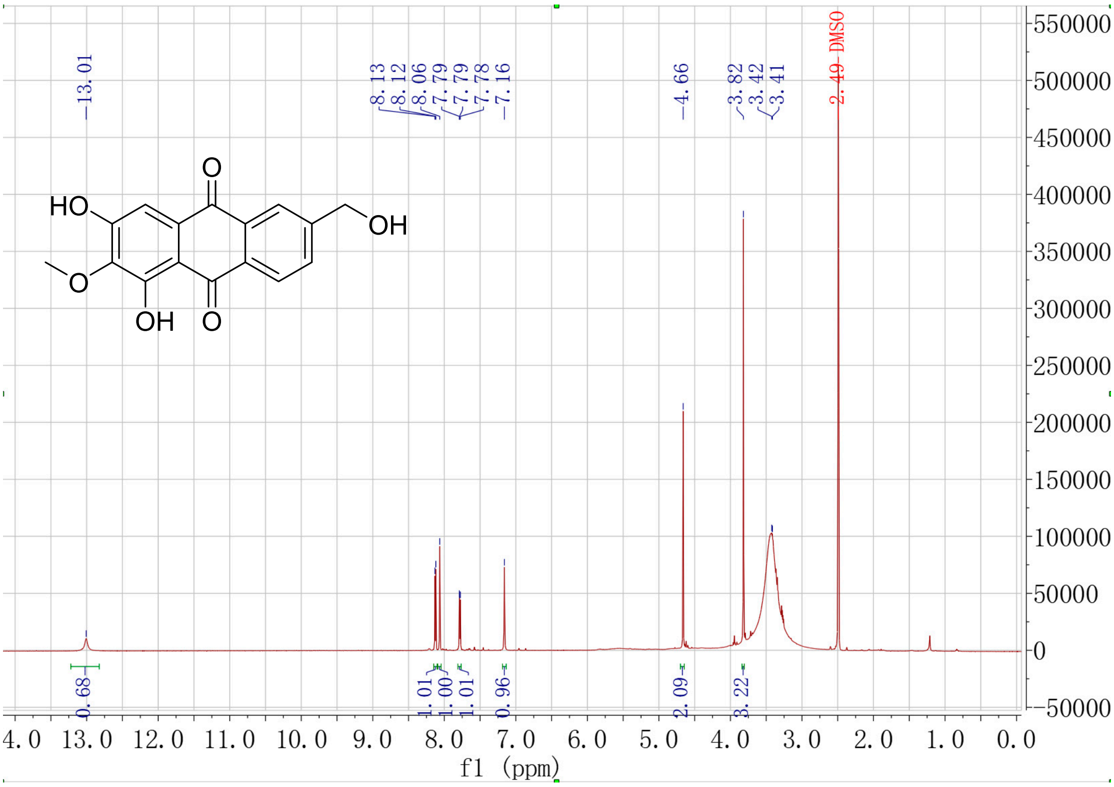

Figure S4-1. ^{1}H NMR spectrum of compound **4**

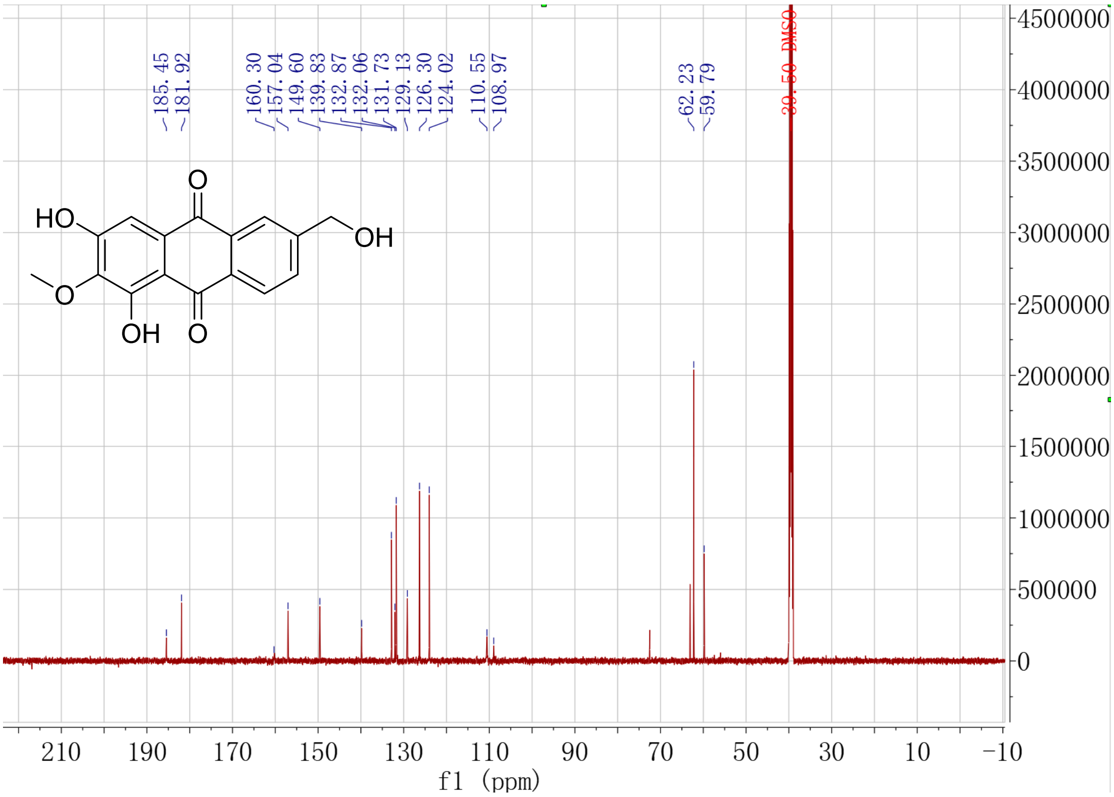

Figure S4-2. ^{13}C NMR spectrum of compound **4**

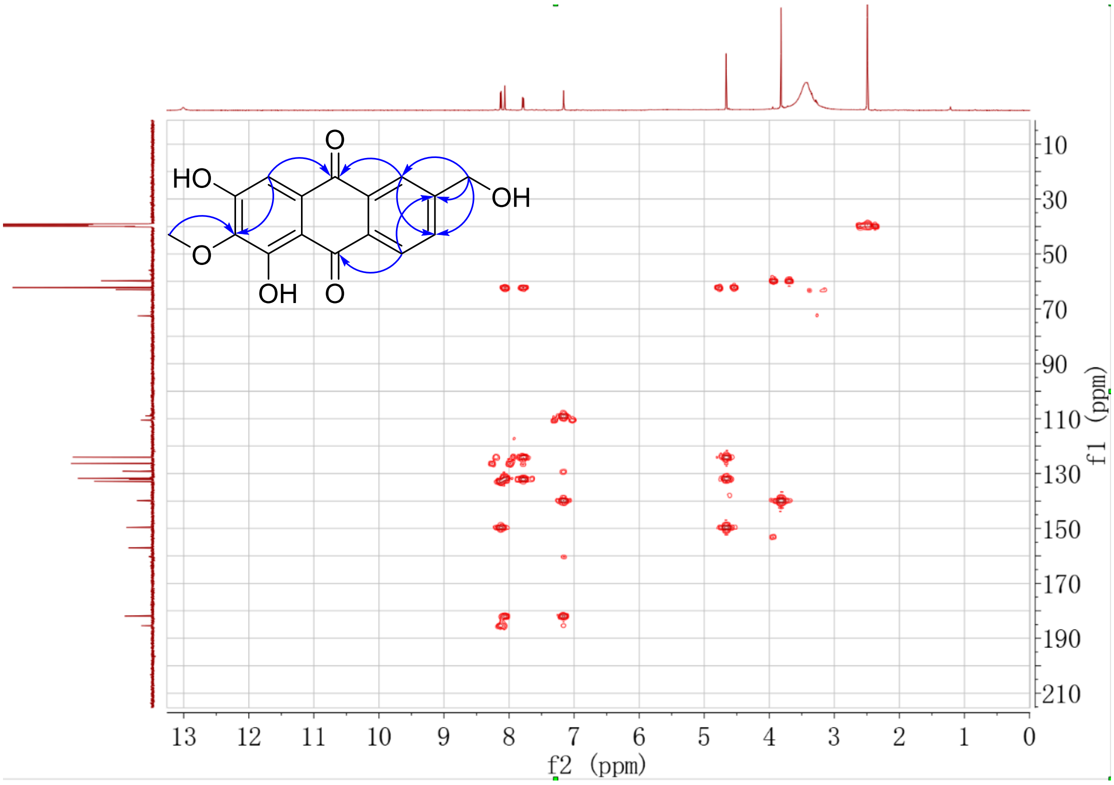

Figure S4-3. HMBC NMR spectrum of compound **4**
